# AI-generated 3D models enhance CBCT interpretation of root canal anatomy among undergraduate and postgraduate students

**DOI:** 10.1007/s00784-026-06897-6

**Published:** 2026-05-07

**Authors:** Rocharles Cavalcante Fontenele, Airton Oliveira Santos-Junior, João Guilherme dos Santos Cunha, Thantrira Porntaveetus, Hugo Gaêta-Araujo, Reinhilde Jacobs

**Affiliations:** 1 Department of Stomatology, Public Health and Forensic Dentistry, Division of Oral Radiology, School of Dentistry of Ribeirão Preto, University of São Paulo (USP), Av. do Café, S/N, Ribeirão Preto, São Paulo 14.040-904 Brazil; 2 OMFS IMPATH Research Group, Department of Imaging and Pathology, Faculty of Medicine, KU Leuven, Leuven, Belgium; 3 Department of Physiology, Faculty of Dentistry, Center of Excellence in Precision Medicine and Digital Health, Chulalongkorn University, Bangkok, Thailand; 4 Department of Dentistry, Universidade da Amazônia (UNAMA), Belém, Pará Brazil; 5 Center of Excellence in Precision Medicine and Digital Health, Chulalongkorn University Implant and Esthetic Center, Faculty of Dentistry, Chulalongkorn University, Bangkok, Thailand; 6 Department of Oral and Maxillofacial Surgery, University Hospitals Leuven, Leuven, Belgium; 7 Department of Dental Medicine, Karolinska Institutet, Stockholm, Sweden

**Keywords:** Endodontics, Educational training, Artificial intelligence, Cone beam computed tomography, Root canal segmentation, Anatomy

## Abstract

**Objectives:**

To evaluate the efficacy of artificial intelligence (AI)-driven three-dimensional (3D) anatomical models as an adjunct to cone-beam computed tomography (CBCT) for root canal assessment regarding diagnostic accuracy, observer confidence, and time efficiency among undergraduate and postgraduate students.

**Materials and methods:**

In this observational diagnostic study, 26 observers (13 undergraduates and 13 postgraduates) evaluated 22 tooth roots with complex anatomy from nine CBCT scans under two conditions: CBCT alone and CBCT supplemented with AI-generated 3D anatomical models. Observers assessed the number of roots, root canals, and apical foramina, while confidence (5-point Likert scale) and assessment time were recorded. Each observer performed 132 assessments, totaling 3,432 evaluations. A reference standard was established by consensus between two specialists. A significance level was set at 5% (α = 0.05) for all statistical analyses.

**Results:**

Augmenting CBCT with AI-generated 3D models significantly improved diagnostic accuracy for all parameters (*p* < 0.001). Root detection accuracy reached 100% in both groups. Root canal detection increased from 83% to 94% among undergraduates and from 88% to 99% among postgraduates, while apical foramina detection increased to 99% in both groups. Observer confidence significantly increased (*p* < 0.001), reaching a median score of 5 (IQR: 5–5). Workflow efficiency also improved (*p* < 0.001), with median assessment time decreasing from 102 s to 39 s for undergraduates and from 97 s to 24 s for postgraduates.

**Conclusion:**

AI-driven 3D anatomical models used with CBCT enhance diagnostic accuracy, observer confidence, and evaluation efficiency in endodontic assessment. However, multi-centre studies with larger, more diverse samples, particularly including cases with pronounced artefacts, would further support generalisability.

**Clinical Relevance:**

AI-generated 3D anatomical models derived from CBCT scans may serve as a valuable adjunct for the interpretation of complex root canal anatomy, improving diagnostic accuracy, increasing observer confidence, and reducing assessment time. These findings support their potential role not only in clinical decision-making but also as an effective educational tool for training dental students and clinicians.

## Introduction

Endodontic treatment remains a significant clinical challenge, largely due to the anatomical complexity and variability of the root canal system. Internal tooth morphology may present ramifications, pronounced curvatures, accessory canals, and other variations that differ substantially among tooth groups [1]. This complexity complicates the identification, instrumentation, and obturation of root canals, requiring not only technical proficiency but also a thorough understanding of root canal anatomy for accurate clinical decision-making, particularly in complex cases [1–5].

Given these challenges, endodontic education must extend beyond conventional teaching methods to promote a three-dimensional (3D) understanding of root canal morphology [6]. Although traditional approaches based on two-dimensional images and videos are useful for foundational learning, they often fail to adequately represent the spatial complexity of internal dental anatomy [7, 8]. This limitation may hinder the development of clinical reasoning and negatively impact students’ performance. Consequently, there is an increasing demand for educational strategies that provide more realistic and didactic visualisation of endodontic structures, better aligning training with clinical practice requirements [6–9].

In this context, artificial intelligence (AI)-driven automated segmentation of pulp cavity structures on cone-beam computed tomography (CBCT) scans has emerged as a promising tool. This technology enables the generation of patient-specific 3D models that can enhance both the teaching-learning process and digital workflows in Endodontics [2–5, 10]. These models accurately reproduce complex anatomical features observed in vivo, including curved, multiple, or narrow root canals [3, 11], allowing students to better visualise and interpret internal anatomy. Moreover, they provide an ethically sound and clinically relevant platform for developing technical skills and improving confidence prior to patient care [6, 9, 11].

Despite these advantages, the integration of such technologies into endodontic education remains limited, and their impact on diagnostic performance and confidence has not been fully established. Addressing this gap is essential, particularly in light of ongoing challenges in traditional education, including variability in training standards, increasing student demand, and the need for more interactive and personalised learning approaches [6, 11–13].

Therefore, the aim of this study was to assess the educational benefit provided by AI-driven 3D endodontic anatomical models, when used in conjunction with CBCT, on diagnostic performance, confidence, and time efficiency in the evaluation of root canal anatomy by undergraduate and postgraduate students. The null hypothesis stated that there would be no significant differences in diagnostic performance, confidence levels, or time efficiency between assessments performed using CBCT alone and those performed using CBCT combined with AI-driven 3D anatomical models.

## Materials and methods

This observational study was approved by the Local Ethics Committee under protocol number 7.738.186 and was conducted in accordance with the principles of the World Medical Association’s Declaration of Helsinki.

### Students’ recruitment

Twenty-six undergraduate and postgraduate students from the Ribeirão Preto School of Dentistry, University of São Paulo (FORP/USP), were included in the study and provided written informed consent. The undergraduate group comprised 13 fourth- and fifth-year students who had completed the Oral Radiology course, as basic knowledge of CBCT assessment was required. Additionally, all participants had previously completed Endodontics courses, including internal root canal morphology. In addition, 13 postgraduate students enrolled in the Restorative Dentistry and Oral Rehabilitation programs at the same institution participated. Basic knowledge of CBCT examination was also required. Similar to the undergraduate group, these postgraduate students had previously completed training in Endodontics.

### Sample selection and preparation

An oral and maxillofacial radiologist with 10 years of experience in CBCT imaging (R.C.F.) retrospectively selected nine CBCT scans from the imaging centre database, comprising 12 teeth with variable and complex root canal anatomy that posed increased diagnostic difficulty relative to the level of expertise of the study participants. The CBCT scans were acquired using OP300 (Instrumentarium Dental, Tuusula, Finland) and PreXion 3D (PreXion Inc., Tokyo, Japan) units. Acquisition parameters varied across scans and included a field of view (FOV) ranging from 4 × 6 to 8 × 6 cm, voxel sizes between 85 and 200 μm, a tube voltage of 90 kilovoltage peak (kVp), and tube current levels ranging from 7 to 10 mA for the OP300 unit. For the PreXion 3D unit, acquisition parameters included a 5 × 5 cm FOV, a voxel size of 100 μm, a tube voltage of 90 kVp, and a tube current of 4 mA.

As shown in Table 1, a total of 12 teeth obtained from 9 CBCT scans were included in the dataset. Among these, 5 teeth had one root, 4 had two roots, and 3 had three roots, resulting in a total of 22 roots. The sample comprised molars and premolars with multiple roots and canals, as well as single-rooted teeth presenting two root canals, including maxillary right first premolar, maxillary left second premolar, mandibular left canine, and mandibular right lateral incisor, to ensure anatomical variability.

**Table 1:** Sample characterization of teeth included in CBCT dataset according to tooth type, number of roots, number of root canals per root, and number of apical foramina per root

CBCT Scan	Tooth	Number of Roots	Number of Root Canals (per root)	Number of Apical Foramina (per root)
#1	Tooth 36	Two	Two at mesial root	One at mesial root
			One at distal root	One at distal root
#2	Tooth 14	Three	One at mesiobuccal root	One at mesiobuccal root
			One at distobuccal root	One at distobuccal root
			One at palatal root	One at palatal root
#3	Tooth 25	One	Two root canals	One apical formamen
	Tooth 33	One	Two root canals	One apical formamen
#4	Tooth 42	One	Two root canals	One apical formamen
	Tooth 43	One	Two root canals	One apical formamen
#5	Tooth 24	Three	One at mesiobuccal root	One at mesiobuccal root
			One at distobuccal root	One at distobuccal root
			One at palatal root	One at palatal root
#6	Tooth 46	Two	Two at mesial root	One at mesial root
			One at distal root	One at distal root
#7	Tooth 15	One	Two root canals	One apical formamen
	Tooth 46	Two	Two at mesial root	Two at mesial root
			Two at distal root	One at distal root
#8	Tooth 37	Two	One at mesial root	One at mesial root
			One at distal root	One at distal root
#9	Tooth 16	Three	Two at mesiobuccal root	One at mesiobuccal root
			One at distobucaal root	One at distobuccal root
			One at palatal root	One at palatal root

Regarding root morphology, two-rooted teeth typically presented mesial and distal roots, whereas three-rooted teeth exhibited mesiobuccal, distobuccal, and palatal roots. Root canal configuration varied both between and within roots, with some roots containing a single canal and others presenting two canals within the same root. At the apical level, the number of apical foramina per root ranged from one to two.

This heterogeneous anatomical distribution was designed to reflect clinically relevant variations encountered in endodontic practice, thereby increasing the diagnostic demands of CBCT-based assessment of root canal anatomy. Teeth were included when they exhibited either a discrepancy between the number of root canals and apical foramina within a given root, indicating canal confluence or division along its course, or a variation in the number of roots compared with the morphology most commonly expected for the respective tooth type.

All anatomical characteristics were established prior to observer assessment and served as the reference standard for evaluating the diagnostic accuracy of the imaging approaches assessed in this study. Reference information was established by consensus between one oral and maxillofacial radiologist (R.C.F.) and one endodontist (A.O.S.J.), each with at least 5 years of experience in CBCT interpretation. Initially, both observers independently evaluated all cases through dynamic navigation of the CBCT scans using dedicated imaging software (OnDemand 3D, Cybermed Inc., Irvine, CA, USA). Subsequently, a consensus meeting was held to compare their assessments and identify any discrepancies. As no disagreements were observed between the two evaluations, interobserver agreement metrics were not calculated.

### Sample preparation

**Fig. 1:**
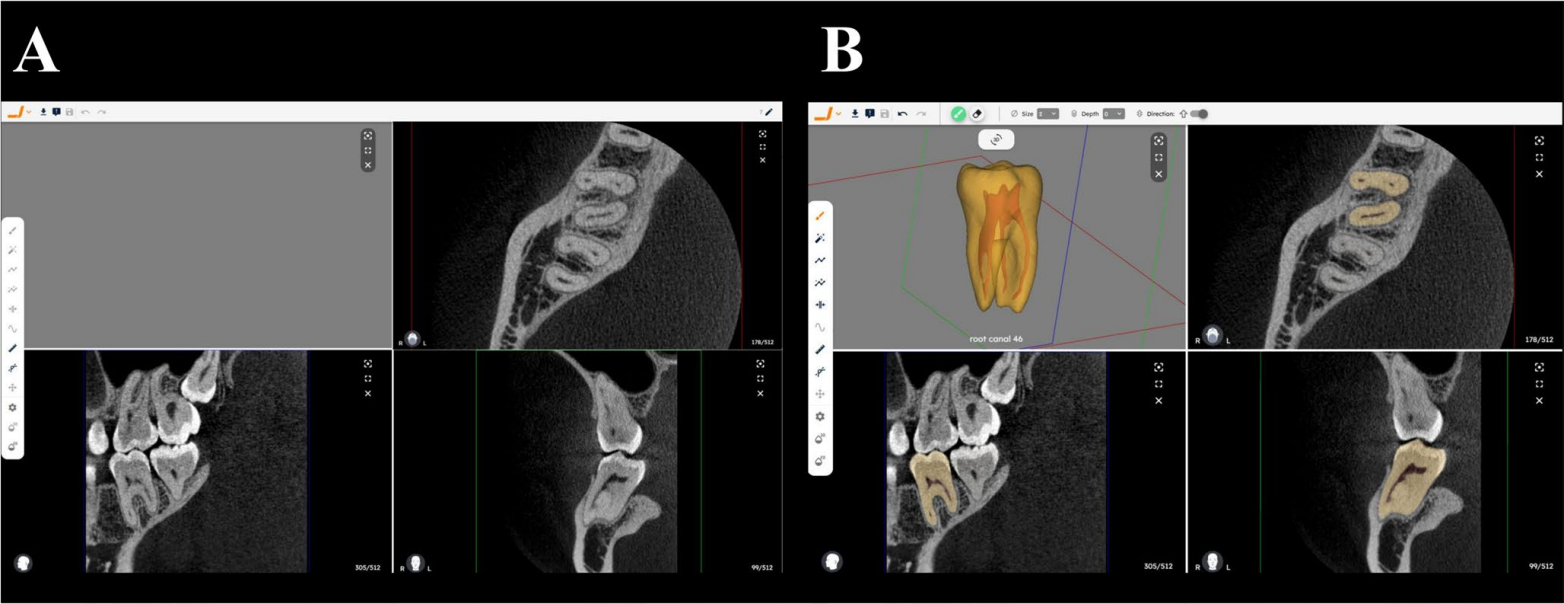
Display of the artificial intelligence (AI)-driven cloud-based platform (creator.relu.eu; Relu, Leuven, Belgium) used according to the imaging assessment approaches: (**A**) conventional CBCT assessment and (**B**) CBCT assessment aided by a three-dimensional AI-generated model. CBCT, cone beam computed tomography

The CBCT datasets (*n* = 9) were uploaded on two separate occasions to a cloud-based platform (creator.relu.eu, Relu, Leuven, Belgium) designed for AI-driven anatomical segmentation of dentomaxillofacial structures, including the pulp cavity system. Cases were presented in a randomized order and evaluated under two assessment conditions (Fig. 1): (1) conventional CBCT interpretation, based on dynamic navigation of the dataset without AI-generated segmentation or 3D reconstruction, in which no 3D model was requested during upload **(**Fig. 1A**)**; and (2) AI-assisted evaluation, in which the same CBCT examinations included automatic segmentation of teeth and the pulp cavity system for all teeth present in the volume, followed by 3D model reconstruction **(**Fig. 1B**)**.

Blinding of observers to the assessment condition was not feasible due to the study design, as the presence or absence of 3D models was immediately apparent upon case opening. To minimize potential recall bias, case presentation order was independently randomized for each assessment condition; although the same CBCT datasets were evaluated twice (with and without AI assistance), their evaluation sequence differed between conditions.

**Fig. 2:**
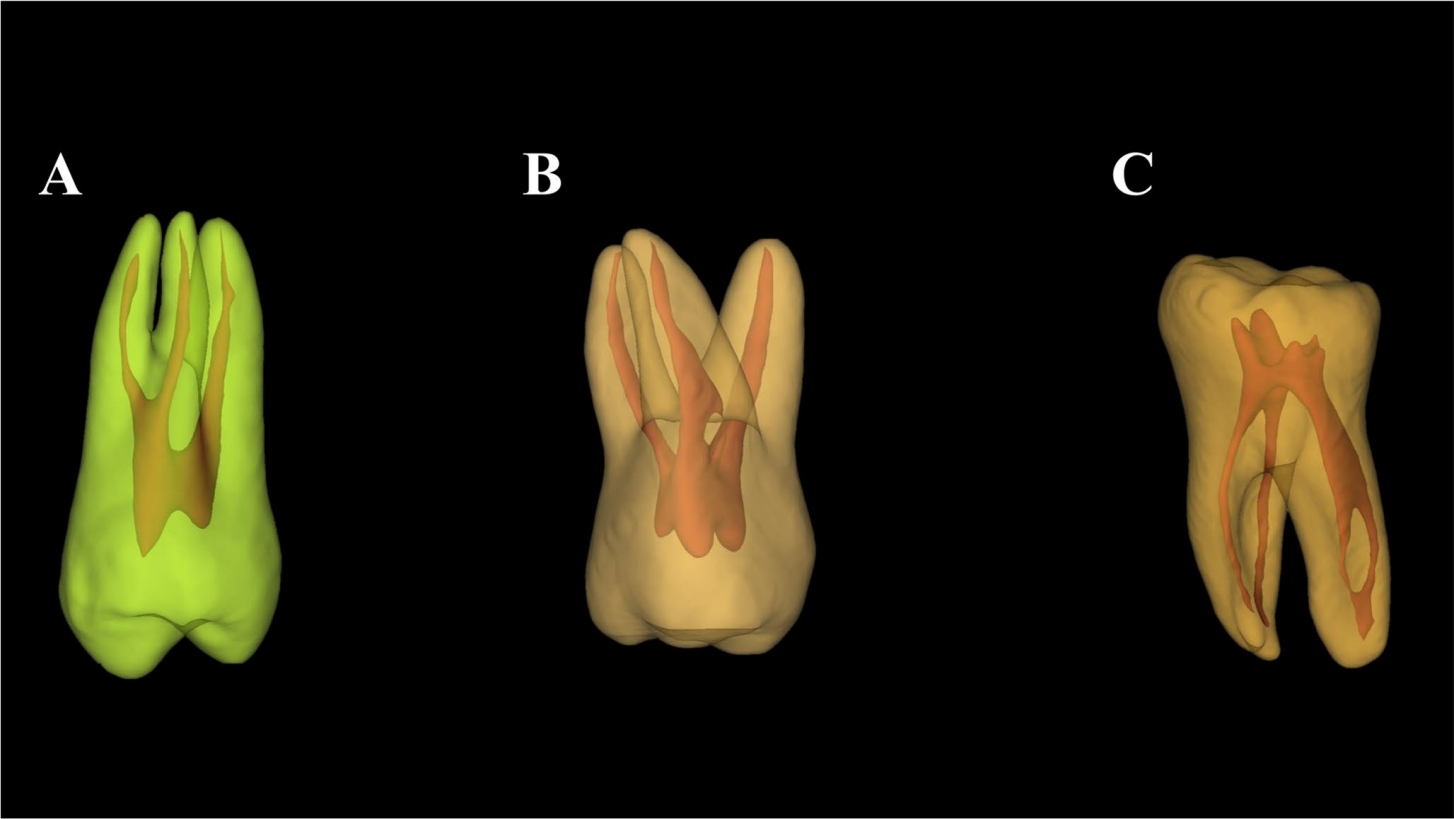
Illustrative three-dimensional artificial intelligence (AI)-generated models representing part of the study imaging sample: (**A**) maxillary right first premolar, (**B**) maxillary right first molar, and (**C**) mandibular right second molar

The AI platform used has been previously validated for tooth and pulp cavity system segmentation in single-rooted, bi-rooted, and multirooted teeth, demonstrating excellent performance, with Dice Similarity Coefficient values ranging from 95% to 99% for tooth segmentation and from 88% to 93% for pulp cavity system segmentation [2, 4, 5, 14]. All automatically generated segmentations were reviewed and verified by one observer (R.C.F.), and no manual refinement was required. Figure 2 illustrates examples of AI-generated 3D tooth models from the study sample.

### Imaging assessment

Before image assessment, undergraduate and postgraduate students participated in a calibration session to standardize evaluation criteria. During this session, the endodontic anatomical structures to be assessed on CBCT scans were explained in detail for each tooth. The calibration session was conducted individually for each observer using two representative CBCT scans not included in the study sample. These cases were selected to illustrate different anatomical configurations and to standardise the assessment parameters (i.e., number of roots, number of root canals per root, and number of apical foramina per root). The first case was an upper left second premolar with one root, two root canals, and a single apical foramen, illustrating canal convergence. The second case was a mandibular right first molar with two roots: the mesial root presented two root canals converging into one apical foramen, while the distal root presented one root canal and one apical foramen. These cases were used to familiarise participants with distinct patterns of root canal anatomy prior to the evaluation.

Observers were also introduced to the cloud-based analysis platform and its tools to ensure adequate familiarization with the interface, including image navigation and multiplanar reconstruction alignment. They were instructed to adjust brightness, contrast, and zoom according to their preferences and to use the resliceable axes tool to realign the axial, sagittal, and coronal planes, allowing the CBCT volume to be oriented along the long axis of each root under examination.

Each observer independently evaluated the cases and recorded, for each predefined tooth on the CBCT scans, the number of roots, the number of root canals per root, and the number of topographically identifiable apical foramina. Both imaging conditions (CBCT alone and CBCT with AI-generated 3D models) were assessed within a single session. Cases were presented in a randomized sequence that interleaved both conditions to minimise recall bias and reduce direct comparison of the same case. Responses were recorded using an online data collection form (Google Forms, Google LLC, Mountain View, CA, USA). For each parameter assessed, observers also reported their level of confidence using a 5-point Likert scale (1 = not confident at all; 2 = slightly confident; 3 = neutral; 4 = moderately confident; 5 = completely confident). The time taken to assess each image was recorded individually for each tooth using a digital stopwatch, for both imaging assessment methods. This was timed from when the observer initiated realignment of the CBCT volume according to the long axis of the root under evaluation, to when the observer felt confident enough to provide responses for the three assessed parameters.

Image assessment was performed on a workstation equipped with an AMD Ryzen 9 9700 × 8-core processor (3.80 GHz), 32 GB of RAM, and an NVIDIA GeForce RTX graphics card, using a 21.5-inch AOC monitor with Full HD resolution (1920 × 1080 pixels), under dimmed-light and silent environmental conditions. For each observer, a total of 132 image assessments were performed (22 tooth roots × 2 imaging approaches × 3 anatomical parameters). As 26 students were involved, a total of 3,432 image assessments were recorded.

### Statistical analysis

Statistical analyses were performed using Jamovi software (version 2.7; Jamovi Project, Sydney, Australia). Diagnostic accuracy was expressed as the proportion of correct responses and reported with 95% confidence intervals, analysed separately for undergraduate and postgraduate students. Comparisons of diagnostic accuracy between CBCT alone and CBCT combined with the AI-driven 3D anatomical model were conducted using the McNemar test.

Confidence levels and the total time required to assess all endodontic anatomical parameters were summarized as medians and interquartile ranges (25th–75th percentiles). Comparisons of both outcomes between imaging conditions were performed using the Wilcoxon signed-rank test. Statistical significance was set at α = 0.05.

The root was considered the unit of analysis due to the variability of root canal morphology within the same tooth. As multiple observations were obtained from the same tooth and from repeated evaluations by the same observers under both imaging conditions, analyses were based on within-observer comparisons, reflecting the paired nature of the data.

A post hoc power analysis was performed using GPower software (version 3.1.9.7; Heinrich Heine University Düsseldorf, Düsseldorf, Germany). Based on the McNemar test and considering the total sample size used in the study, a statistical power greater than 99% was achieved to detect the observed difference in diagnostic accuracy between CBCT alone and CBCT combined with the AI-driven 3D anatomical model (effect size w = 0.24, α = 0.05).

## Results

### Diagnostic accuracy

**Fig. 3:**
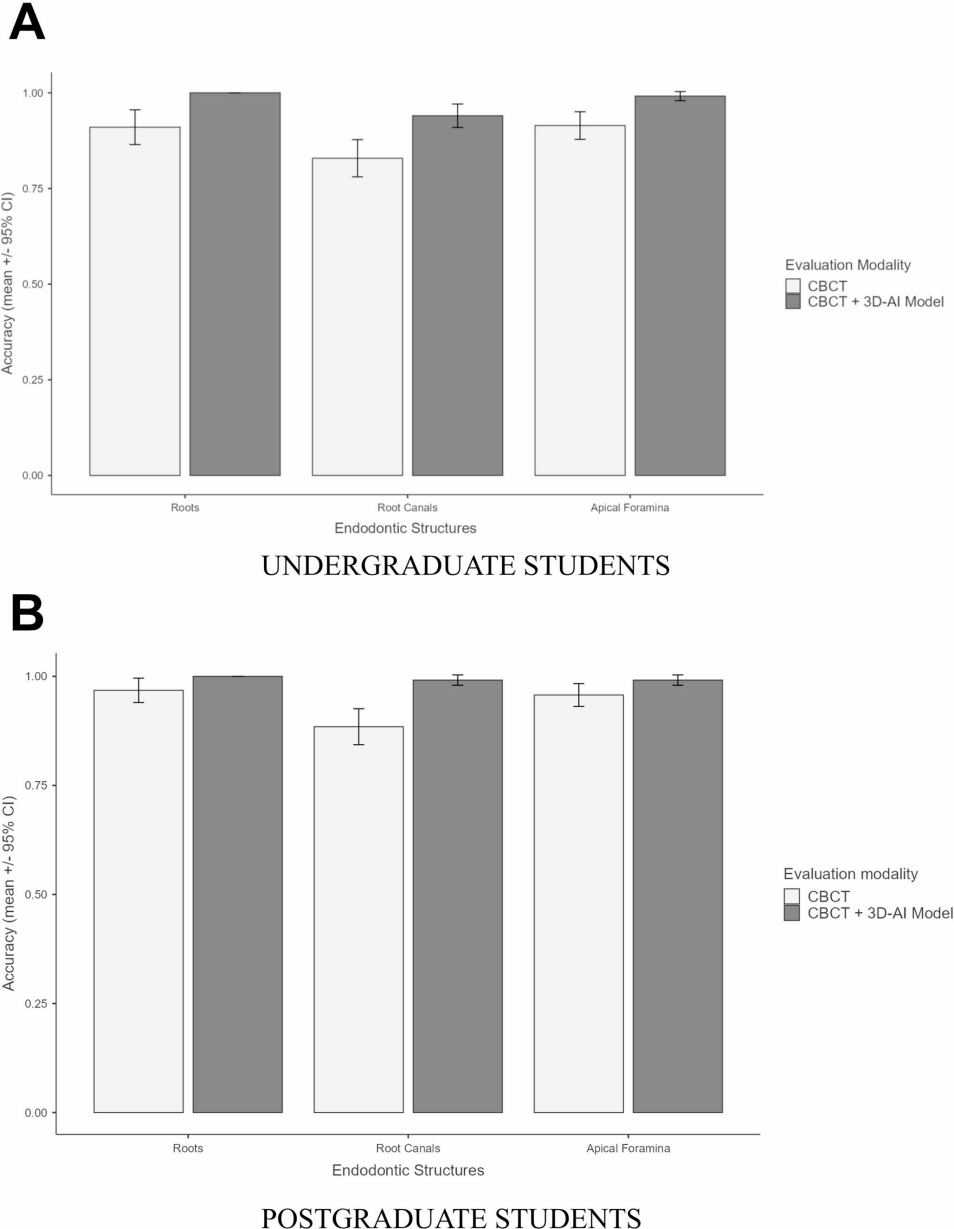
Bar charts showing diagnostic accuracy (mean percentage of correct responses with 95% confidence intervals) for the assessment of root canal anatomy parameters using each imaging approach: (**A**) undergraduate students and (**B**) postgraduate students

Diagnostic accuracy for the assessment of root canal anatomy parameters is summarized in the bar graphs shown in Fig. 3. Compared with CBCT alone, the combination of CBCT with the AI-driven 3D anatomical model resulted in significantly higher diagnostic accuracy for all evaluated parameters (*p* < 0.001). This improvement was observed in both undergraduate (*p <* 0.001) and postgraduate students (*p <* 0.05).

For the assessment of the number of roots, the proportion of correct responses among undergraduate students increased significantly from 91% (95% CI: 86.5–95.6%) with CBCT alone to 100% (95% CI: 100–100%) with 3D-AI–assisted assessment (*p* < 0.001). A similar improvement was observed among postgraduate students, with accuracy increasing from 97% (95% CI: 94–100%) to 100% (95% CI: 100–100%) (*p* = 0.04).

Regarding the number of root canals, diagnostic accuracy improved significantly with the use of the 3D-AI model for both undergraduate students (83% vs. 94%, *p* < 0.001) and postgraduate students (88% vs. 99%, *p* < 0.001).

A similar trend was observed for the assessment of the number of apical foramina. The use of the AI-driven 3D model significantly increased (*p* < 0.001) the proportion of correct responses among undergraduate students from 91% (95% CI: 87.8–95.1%) to 99% (95% CI: 95.6–100%). Among postgraduate students, accuracy also increased (*p* < 0.001) from 95.7% (95% CI: 98–100%) to 99% (95% CI: 99–100%).

### Confidence level

**Fig. 4:**
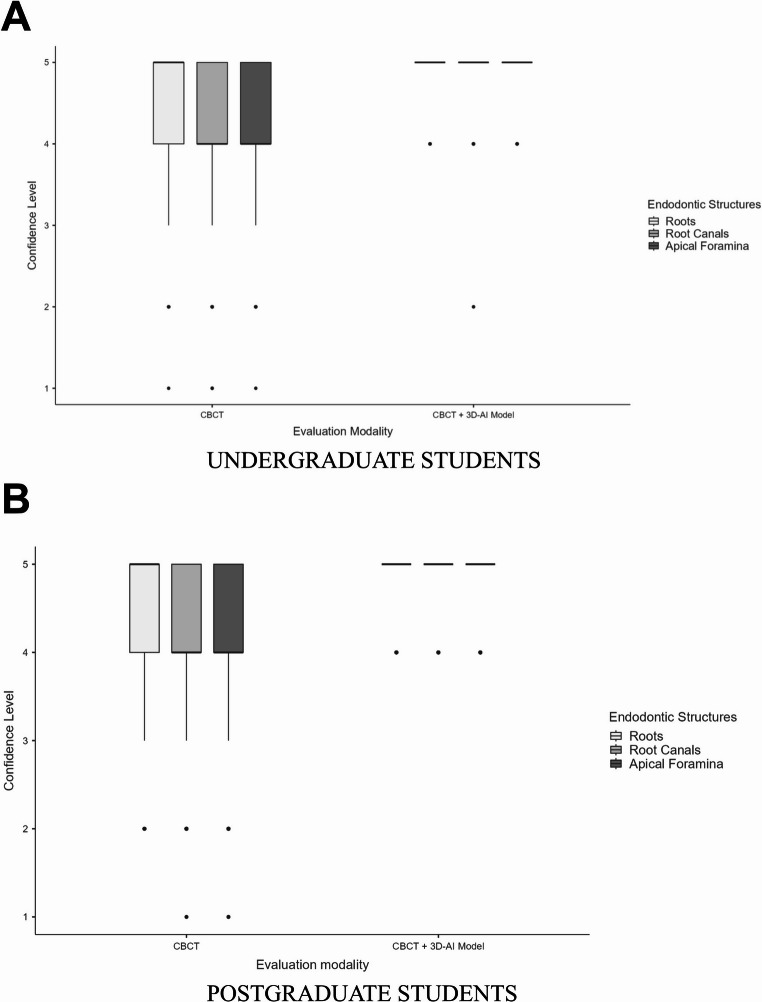
Box plots showing median confidence levels (25th – 75th percentiles) for the assessment of root canal anatomy parameters within each imaging approach tested: (**A**) undergraduate students and (**B**) postgraduate students

Participants’ confidence levels for the assessment of root canal anatomy parameters are illustrated in the box plots presented in Fig. 4. The use of the AI-driven 3D anatomical model in combination with CBCT resulted in significantly higher confidence levels compared with CBCT alone for all evaluated parameters (*p* < 0.001).

For the assessment of the number of roots, median confidence scores differed significantly between imaging conditions. For both undergraduate and postgraduate students, confidence scores changed from a median of 5 (IQR: 4–5) with CBCT alone to 5 (IQR: 5–5) with CBCT+3D-AI model (*p* < 0.001).

Regarding the assessment of the number of root canals and apical foramina, the availability of the AI-driven 3D model led to a significant increase in confidence levels for both undergraduate and postgraduate students. In both groups, median confidence scores increased from 4 (IQR: 4–5) with CBCT alone to 5 (IQR: 5–5) with CBCT+3D (*p* < 0.001).

### Timing analysis

**Fig. 5:**
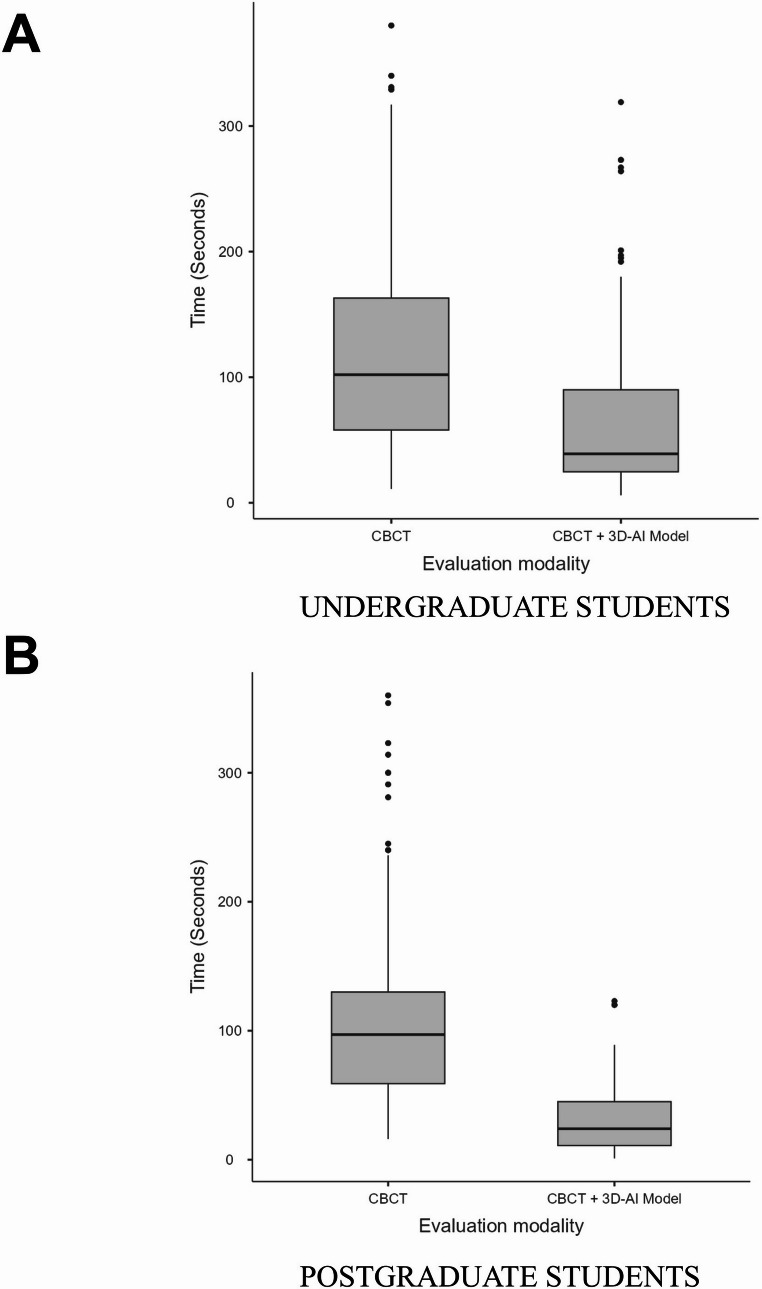
Box plots showing median time required levels (25th – 75th percentiles) for the assessment of all endodontic anatomical parameters within each imaging approach: (**A**) undergraduate students and (**B**) postgraduate students

Figure 5 presents box plots illustrating the time required to assess endodontic structures (number of roots, root canals, and apical foramina) within each assessment modality. For both undergraduate and postgraduate students, the time required to assess endodontic structures was significantly reduced when the AI-driven 3D model was used in combination with CBCT compared with CBCT alone (*p* < 0.001).

Among undergraduate students, the median assessment time decreased significantly from 102 s (IQR: 58–163 s) with CBCT alone to 39 s (IQR: 24.8–90 s) when the 3D model was available (*p* < 0.001). Similarly, postgraduate students demonstrated a significant reduction in assessment time when CBCT was combined with the AI-driven 3D model, with the median time decreasing from 97 s (IQR: 59–129 s) with CBCT alone to 24 s (IQR: 11–45 s) with CBCT+3D (*p* < 0.001).

## Discussion

This observational study examined whether integrating AI-driven 3D anatomical models with CBCT imaging enhances students’ interpretation of root canal anatomy across different levels of training, addressing a relevant and still underexplored gap in endodontic education. While prior studies have suggested that AI-based 3D visualization may facilitate anatomical understanding [2–5, 15], evidence of its concrete effects on diagnostic accuracy, confidence, and time-efficiency in CBCT-based evaluations within endodontic education is still lacking. The present results demonstrate that AI-generated 3D models consistently improved diagnostic accuracy for key anatomical parameters, increased students’ confidence levels, and significantly reduced assessment time when compared with CBCT alone. Notably, these benefits were observed in both undergraduate and postgraduate students, indicating that the impact of AI-assisted 3D visualization extends across different stages of professional training. In combination, these findings indicate that this approach provided objective gains in diagnostic performance and workflow efficiency rather than merely enhancing subjective perception. Therefore, the null hypothesis was rejected, as significant differences were identified between conventional CBCT assessment and CBCT combined with AI-driven 3D anatomical models.

Within this educational context, the incorporation of an AI-driven 3D anatomical model into CBCT-based assessment was associated with improved diagnostic accuracy, supporting its potential role as an adjunct for learning root canal anatomy [16–18]. The AI-based online platform used to generate the anatomical models has been evaluated in previous studies [2, 4, 5], which supports its use as a learning tool. However, it is important to note that the present study did not directly assess segmentation accuracy. In this investigation, the generated models were used as provided by the platform, and no additional manual refinement was deemed necessary prior to the assessments. Therefore, the findings should be interpreted in terms of the educational performance associated with the use of the models, rather than as a validation of the underlying segmentation platform.

Across all evaluated anatomical parameters, the availability of the AI-driven 3D model led to significantly higher proportions of correct responses compared with CBCT alone, and this improvement was consistently observed regardless of the participants’ level of training. From an educational perspective, these findings suggest that the AI-driven anatomical models may support anatomical interpretation across different levels of clinical experience, functioning as an intuitive and didactic aid during CBCT evaluation. By presenting volumetric imaging data in an interactive 3D format, the model may facilitate spatial understanding and contribute to more efficient and confident interpretation within an endodontic educational setting.

In this investigation, three clinically important parameters of root anatomy were evaluated to provide further insight into the educational contribution of the AI-driven 3D model to endodontic training: the number of roots, the number of root canals per root, and the number of apical foramina per root. For the assessment of the number of roots, diagnostic accuracy was already high when CBCT was used alone, and the incorporation of the AI-based 3D model led to maximal accuracy among both undergraduate and postgraduate students.

A significant improvement was observed in the assessment of the number of root canals, a task that represents one of the greatest cognitive challenges in endodontic diagnosis [19]. This difficulty arises from the complex, irregular, and highly variable internal morphology of the root canal system [2–5, 19]. Although CBCT provides 3D volumetric data, anatomical interpretation is commonly performed through the sequential evaluation of axial, sagittal, and coronal reconstructions, which increases cognitive demand and reliance on mental spatial integration [20, 21]. In this context, the interactive and dynamic nature of the AI-driven 3D model mitigates this limitation by allowing students to visualize and explore the tridimensionality of the root canal system as a single anatomical structure, rather than across multiple orthogonal imaging planes [22, 23].

A similar, though less pronounced, improvement was also observed in the identification of apical foramina, structures that are particularly difficult to recognize because of their small dimensions and marked anatomical variability [1, 24]. This added value is particularly relevant given known limitations of CBCT imaging, in which grayscale values are not standardised as true Hounsfield units and are sensitive to acquisition and reconstruction parameters, including grayscale threshold selection, which may increase interpretative variability [25, 26]. The AI-generated 3D models used in this study were developed using expert annotations in previous investigations [2, 4, 5] and may provide learners with a more consistent and intuitive visual reference to support anatomical interpretation.

Overall, these findings demonstrate that the AI-driven 3D model enhances diagnostic accuracy by reducing cognitive load and improving spatial understanding, thereby providing a meaningful educational advantage in the interpretation of different aspects of the root canal anatomy. In addition, the present results demonstrated that the use of an AI-driven 3D anatomical model in combination with CBCT was associated with higher confidence levels during the assessment of root canal anatomy.

In endodontic education, confidence is closely related to the ability to interpret anatomical information and to make consistent clinical judgements, particularly in situations involving the anatomical complexity of the root canal system [6, 9, 11, 17]. In the present study, confidence scores were significantly higher for all evaluated parameters in both undergraduate and postgraduate students when the AI-driven 3D model was available. This finding indicates that 3D visualization contributes not only to diagnostic performance, but also to a more secure interpretation of endodontic anatomy. From an educational standpoint, greater confidence may favour more structured clinical reasoning, reduce uncertainty during image analysis, and support a more consistent transition from radiographic interpretation to clinical planning [17].

When confidence was analysed according to anatomical parameters, distinct patterns emerged that further clarify the educational contribution of the AI-driven 3D model. For the assessment of the number of roots, confidence was already high when CBCT was used alone and became consistently maximal with the addition of the AI-based 3D model among both undergraduate and postgraduate students. This pattern suggests a stabilizing effect, reinforcing certainty in tasks that are relatively familiar during endodontic training. In contrast, confidence increased more markedly for the assessment of root canals and apical foramina, with median scores shifting from 4 to 5 in both groups. While these parameters are commonly associated with greater uncertainty during endodontic training, the AI-based 3D model allows a more consistent interpretation of complex anatomical features. Similar findings have been reported in a previous study, in which AI-based segmentation and 3D modelling increased observer confidence and reduced assessment variability [15].

The time required to interpret CBCT scans represents an important component of the learning process in endodontics [11]. Although CBCT scans provide detailed volumetric information, image interpretation is often time-consuming, particularly for students who must analyse complex anatomical features through subsequential axial, sagittal, and coronal reconstructions [20]. The use of the AI-driven 3D anatomical model led to a marked reduction in median assessment time for both undergraduate and postgraduate students. Among undergraduate students, median assessment time decreased from 102 s with CBCT alone to 39 s when CBCT was combined with the AI-based 3D model, corresponding to an approximate 2.6-fold reduction. An even greater positive effect was observed among postgraduate students, in whom median assessment time decreased from 97 s to 24 s, representing an approximate 4.0-fold reduction. These findings indicate that AI-based 3D visualization substantially streamlines the interpretative process, allowing learners to reach accurate conclusions more efficiently, confidently, and rapidly. Importantly, this improvement in time efficiency was achieved without compromising diagnostic accuracy, reinforcing the added educational value of the AI-driven 3D model in supporting a more effective and structured learning experience during CBCT-based endodontic training.

From an educational perspective, this gain in time efficiency is particularly relevant. By reducing the time spent navigating tomographic datasets, students at different stages of endodontic training can allocate greater cognitive resources to anatomical understanding and clinical reasoning. Although some learning effect due to repeated exposure cannot be fully excluded, this is inherent to observer-based assessment tasks. To minimise this effect, both imaging conditions were evaluated within a single session, and cases were presented in a randomised sequence that interleaved CBCT-only and CBCT with AI-generated three-dimensional models, reducing recall bias and limiting direct comparison of the same case across conditions. The single-session design also ensured that all observers were assessed under identical conditions, avoiding variability related to scheduling, environmental factors, and day-to-day fluctuations that could influence performance and time measurements (e.g., variations in internet connection affecting the responsiveness of the AI platform). As all observers followed the same protocol, any residual learning or fatigue effects would have been similarly distributed across both conditions.

In the present study, direct comparisons between undergraduate and postgraduate students were not performed, as the primary objective was not to contrast levels of expertise. Rather, the study aimed to assess whether the addition of AI-driven 3D models provided a consistent educational benefit within each training level. Improvements in diagnostic accuracy, confidence, and assessment time were observed in both groups, indicating that AI-generated 3D models may support CBCT-based anatomical interpretation across different stages of endodontic training. Nevertheless, several limitations should be considered.

CBCT scans presenting a high degree of artefacts were excluded, and cases involving more than one tooth with extensive coronal and/or root filling materials within the same sextant were not included in the dataset. As a result, the interpretative challenge may have been lower than that encountered in clinical scenarios characterised by pronounced beam hardening and blooming artefacts, as previously reported [14]. Therefore, the present findings should not be extrapolated to highly artefact-affected imaging conditions or to non-endodontic anatomical structures. In addition, endodontic specialist trainees were not included, as this study focused on undergraduate students and non-specialist postgraduate trainees within a general educational context. Given the higher level of clinical experience and different learning objectives in specialist training, future studies should investigate the applicability of this approach in specialist-level programmes. Additionally, future investigations are encouraged to evaluate the performance of AI-driven 3D models under more challenging imaging conditions, including the presence of restorative materials, the use of different CBCT devices, and a broader range of anatomical scenarios.

Furthermore, the root was considered the unit of analysis to capture variations in root canal morphology within the same tooth. As multiple observations were obtained from the same tooth and from repeated evaluations by the same observers, the data have a clustered structure. Although paired comparisons were used to evaluate differences between imaging assessment conditions, some degree of non-independence may remain, which could have resulted in lower p-values and narrower confidence intervals. Accordingly, the findings should be interpreted with caution, with greater emphasis placed on the consistency and magnitude of the observed effects rather than on statistical significance alone. Future studies may benefit from mixed-effects modelling to further account for clustering at the observer and tooth/root levels.

The present findings contribute to the ongoing discussion on the use of AI in endodontic education by providing initial evidence that AI-driven 3D anatomical models may add educational value without altering the fundamental principles of CBCT interpretation. Rather than replacing tomographic analysis or clinical reasoning, AI appears to function as an adjunctive educational tool that may support anatomical understanding, enhance learner confidence, and improve efficiency when interacting with CBCT data [17]. However, these implications should be interpreted with caution, as this was a single-centre study based on a relatively small and selected sample, including cases with limited beam-hardening artefacts and specific anatomical characteristics, which may restrict the generalisability of the findings.

Within an educational landscape characterised by increasing technological complexity, AI-driven 3D models of the root canal system may assist educators in standardizing learning experiences, facilitating the interpretation of complex endodontic anatomy, and better preparing students and residents for the cognitive demands of contemporary endodontic practice. At the same time, the present findings underscore the need for a stepwise and critical integration of AI into education, in which human judgment, contextual interpretation, and clinical responsibility remain central [17]. Within this workflow, AI-driven 3D models should be regarded as transparent and structured educational aids that complement conventional CBCT interpretation, contributing to the development of clinicians who are both technically competent and diagnostically confident.

## Conclusion

The use of AI-driven 3D models alongside CBCT was associated with improved diagnostic accuracy in assessing root canal anatomy, as well as higher confidence and reduced interpretation time among both undergraduate and postgraduate students. These findings suggest potential for AI-generated models as an adjunct in endodontic training. However, multi-centre studies with larger, more diverse samples, particularly including cases with pronounced artefacts, would further support generalisability.

## Data Availability

The data that support the findings of this study are available from the corresponding author upon reasonable request.
